# Antipsychotic Use in Bipolar Disorder: Clinical and Genomic Correlates– A Mayo Clinic Bipolar Disorder Biobank Study

**DOI:** 10.21203/rs.3.rs-7274612/v1

**Published:** 2025-08-05

**Authors:** Balwinder Singh, Ada Man-Choi Ho, Brandon J. Coombes, Francisco Romo-Nava, David J. Bond, Marin Veldic, Richard S. Pendegraft, Anthony Batzler, Alfredo B. Cuellar-Barboza, Manuel Gardea-Reséndez, Miguel L. Prieto, Aysegul Ozerdem, Susan L. McElroy, Joanna M. Biernacka, Mark A. Frye

**Affiliations:** Mayo Clinic; Mayo Clinic; Mayo Clinic; Lindner Center of HOPE; Johns Hopkins University; Mayo Clinic; Mayo Clinic; Mayo Clinic; Mayo Clinic; Mayo Clinic; Mayo Clinic; Mayo Clinic; Lindner Center of HOPE; Mayo Clinic; Mayo Clinic

**Keywords:** Bipolar disorder, antipsychotics, treatment response, pharmacogenomics

## Abstract

**Background:**

Responsiveness to mood-stabilizing pharmacotherapy varies in bipolar disorder (BD). We investigated clinical correlates of second-generation antipsychotic (SGA) treatment response and conducted the first genome-wide association study (GWAS), including exploratory polygenic scores (PGS), of SGA pharmacogenomic treatment response in BD.

**Methods:**

Treatment response was quantified using the Alda scale, and GWAS was performed using Alda-A score, controlling for sex, genotyping batch, and the genomic principal components.

**Results:**

The cohort included 2,159 adults with BD (1,416 BD-I, 691 BD-II, 51 schizoaffective BD), mean age 41.8 years, 62% female, 84% white, and 14% Hispanic. Nearly half (48%) were treated with SGAs. Current SGA users were younger (41.2±14.7 vs. 42.5±15.3 years, *p*=0.040), more likely to be Hispanic (14% vs. 11%, *p*=0.047), had a higher body mass index (BMI; 30.4±7.6 vs. 29.5±7.1 kg/m^2^, *p*=0.005). Lifetime comorbidity patterns for current SGA users include higher rates of manic psychosis (29% vs. 17%, *p*<0.001) and eating disorders — Anorexia Nervosa (7% vs. 4%, *p*=0.003), Bulimia Nervosa (7% vs. 4%, *p*=0.003), and Binge Eating Disorder (14% vs. 11%, *p*=0.030). We detected a genome-wide significant association between SGA Alda-A scores and *GAS7* variants (top variant: rs202127418, *β*=−2.998, *p*=4.96E-08). However, SGA response was not significantly associated with PGS for schizophrenia, BD, and major depression (FDR>0.05).

**Conclusions:**

SGAs are frequently utilized as mood stabilizers in patients with BD and are associated with manic psychosis and eating disorders. *GAS7* variants may predict SGA response, but larger, more diverse cohorts are needed for validation.

## INTRODUCTION

Pharmacotherapy plays a crucial role in mood stabilization for bipolar disorders (BD) (Singh et al., 2025). Recent data highlight that second-generation antipsychotics (SGA) are the most commonly prescribed pharmacotherapeutic treatment for BD in outpatient settings (Rhee et al., 2020; Singh et al., 2024). However, predictors associated with SGA prescribing in BD remain understudied (Post et al., 1998). While genome-wide association studies (GWAS) have examined antipsychotic efficacy in schizophrenia (SCZ) (Allen and Bishop, 2019; De Pieri et al., 2024; Fortinguerra et al., 2019; Koromina et al., 2020; Zhang and Malhotra, 2018), no GWAS to date has specifically investigated the efficacy of SGA in BD. Although many pharmacogenomic studies have focused on SCZ and included various first- and second-generation antipsychotics, they offer limited insights into potential genetic markers for antipsychotic response in BD (Fortinguerra et al., 2019; Zhang and Malhotra, 2018).

GWAS have identified significant associations between variants in *CNTNAP5, GRM7*, and *KCNK9* and antipsychotic efficacy in SCZ; these genes have also been identified in GWAS of BD (Fortinguerra et al., 2019). Polygenic scores (PGS) have been applied to study the relationship between genetic liability to certain phenotypes and antipsychotic treatment response. A recent study found that PGS for SCZ was positively correlated with prescribed antipsychotic dose and antipsychotic polypharmacy in five large cohorts of patients with SCZ, BD, and other psychosis (Koch et al., 2024). Another study developed a PGS for antipsychotic response based on 11 single-nucleotide polymorphisms (SNPs) and found a significant association between this PGS and antipsychotic responder status in the overall cohort consisting of patients with SCZ, schizoaffective disorder, and BD. Still, this association was not significant in the BD-only subgroup (De Pieri et al., 2024). Nevertheless, using PGS may help clarify the genetic contributions to SGA response, thereby improving treatment outcome prediction.

This study aimed to investigate clinical correlates of antipsychotic use, particularly SGA, for the treatment of BD. We also investigated potential biological correlates through an exploratory GWAS and PGS analysis of SGA treatment response in BD.

## METHODS

Data for this study were collected from adult individuals (18–80 years) with BD who were enrolled in the Mayo Clinic Bipolar Disorder Biobank (MCBDB) (Frye et al., 2015). Established in July 2009, the MCBDB enrolled patients at five sites: Mayo Clinic, Rochester, Minnesota; Lindner Center of HOPE/University of Cincinnati College of Medicine, Cincinnati, Ohio; the University of Minnesota, Minneapolis, Minnesota; Universidad Autónoma de Nuevo León, Mexico; and Universidad de los Andes, Chile. The Mayo Clinic Institutional Review Board approved the study. Detailed information about the MCBDB has been published previously (Frye et al., 2015; Gardea-Resendez et al., 2022; Pahwa et al., 2021a). In brief, the MCBDB was created to identify biological risk factors for BD, as well as clinical and biological factors associated with BD sub-phenotypes, prognosis, and treatment outcomes. Participants needed to be English-speaking at the U.S. sites and Spanish-speaking at the Mexico and Chile sites, able to provide written informed consent, and meet DSM-IV-TR criteria for BD-I/BD-II or schizoaffective disorder, bipolar type. Patients with active psychosis or active suicidal ideation were excluded.

Medication data, demographics, psychiatric (adult attention deficit hyperactivity disorder [ADHD], childhood ADHD, anorexia nervosa [AN], bulimia nervosa [BN], binge eating disorder [BED], generalized anxiety disorder [GAD], obsessive compulsive disorder [OCD], panic disorder, posttraumatic stress disorder [PTSD], and social anxiety disorder) and medical comorbidities, and family history were collected at the time of study enrollment. The Eating Disorder Diagnostic Scale (EDDS) score was used to measure the eating disorder symptomatology (Stice et al., 2000). To assess the medical illness burden of the study patients, we used the Modified Cumulative Illness Rating Scale (MCIRS) (Salvi et al., 2008), which includes 14 items across various systems, with each item scored from 0 to 4. The severity index is calculated as the mean score of the first 13 categories — excluding psychiatric comorbidity — to evaluate the overall comorbidity burden. Individual categories mean scores were analyzed to examine specific comorbidities.

We collected data on the current and lifetime prescriptions for antipsychotics, mood stabilizing anticonvulsants (MSAC), lithium, and antidepressants. The FGA prescribed in the study cohort included chlorpromazine, fluphenazine, haloperidol, perphenazine, thiothixene, and trifluoperazine. The SGA prescribed included aripiprazole, clozapine, olanzapine, paliperidone, pimozide, quetiapine, ziprasidone, and risperidone. Because the rate of FGA prescriptions was low in the full sample (*n* = 16), our analysis focuses on clinical and genetic predictors of SGA response and current use.

### Measurement of SGA treatment response by the Alda scale

We assessed the response to SGA using the Alda Scale modified for non-lithium mood stabilizing treatments (Grof et al., 2002). This scale was originally developed to retrospectively evaluate prophylactic treatment response to lithium under naturalistic conditions and has since also been used to assess responses to other mood stabilizers (Cuellar-Barboza et al., 2020; Ho et al., 2020). It includes two subscales: Subscale A measures clinical improvement in severity, duration, and frequency of illness, rated from 0 to 10, while Subscale B evaluates five potential confounders of response including the number and frequency of episodes off treatment, duration of treatment, treatment compliance during stable period, and the use of additional medications during the stable period. For clinical analysis, we classified patients with BD who had used SGA and provided treatment response data into SGA responders (A score ≥ 7) and nonresponders (A score < 7) for comparisons. For genetic analysis, we used the A score as a continuous variable to maximize sample size. Because multiple SGA can be assessed with the Alda scale for a single participant, we preferentially chose the A score for the SGA with lowest B score (more confidence in A score). In the case that multiple SGA had the same B score, we averaged the A scores to obtain a combined SGA response outcome. This approach to combining Alda scores across medications has been previously used when assessing MSAC (Ho et al., 2020). The Alda A score has shown comparable intra-class correlation to the total score, while Subscale B is more prone to measurement errors (Manchia et al., 2013; Scott et al., 2019). This methodology has been employed in previous studies (Joseph et al., 2023; Pahwa et al., 2021b).

### Statistical analysis of demographics and clinical factors

We first aimed to identify demographic and clinical correlates associated with current SGA prescriptions in patients with BD, as well as correlates of SGA treatment response. To achieve this, we first performed comparisons of demographic and clinical variables: (1) between participants with BD currently on an SGA and those not on any antipsychotics, (2) among participants currently on one SGA and those currently on more than one SGA, and (3) between SGA responders (Alda A score ≥ 7) and non-responders (Alda A score < 7). Additionally, we explored differences between participants with BD currently using an SGA versus an FGA. ANOVA and Pearson’s chi-squared test were used for comparisons of continuous and categorical variables, respectively. We do not report tests of significance for any variable with an observed frequency less than 5%. Statistical analysis was performed using R 4.2.2. To adjust for multiple comparisons in these analyses, we use a significance threshold of *p* < 0.001 to identify meaningful differences.

### Genotyping, imputation, and GWAS

Genotyping and imputation of MCBDB samples from Mayo Clinic patients were conducted using Genotyping-by-Sequencing (GxS) technology through the Regeneron Genetics Center (Tarrytown, NY, USA), with a detailed methodology published previously (Gelfman et al., 2023). For participants enrolled at other sites, genotyping was performed with either the Illumina HumanOmniExpress 12v1 or the Global Screening Array 24v2. Within each batch, samples were excluded for inconsistencies in sex, elevated genotype missingness (>5%), or heterozygosity below 70% on multiple chromosomes. Variants were excluded if they exhibited high missingness (>0.5%), low minor allele frequency (MAF < 0.05), or deviated from Hardy-Weinberg equilibrium (*p* < 1E-06).

Each batch was subsequently imputed via the TOPMed imputation server (Taliun et al., 2021). Post-imputation, batches were merged, retaining only variants with a dosage-R2 > 0.3 across all batches. FlashPCA (Abraham et al., 2017; Alexander and Lange, 2011) was used to calculate principal components (PCs) for each subject. KING software evaluated relatedness, and for any pair with a second-degree or higher relatedness (kinship coefficient ≥ 0.0442), one individual was randomly removed (Manichaikul et al., 2010).

For the GWAS of SGA response, we used linear regression to assess the association between each SNP (in terms of allele dosage) and Alda A score as a continuous variable. We adjusted for sex, genotyping batch, and the first three PCs of ancestry. In total, 517 participants were included in the GWAS analysis. GWAS results were annotated by the human genome assembly GRCh38 for genomic location and nearby genes. SNP associations with *p* < 5E-08 were considered statistically significant. Analyses were run using PLINK2 (Chang et al., 2015).

### Polygenic score analysis

Polygenic scores (PGS) were computed using LDpred2-auto in the *bigsnpr* R package (Prive et al., 2023; Prive et al., 2021). Only SNPs in Hapmap3+ with a MAF > 5% and a dosage-R2 > 0.8 were included. PGS were computed using summary statistics for BD, major depressive disorder (MDD), and SCZ, and were standardized to a mean of 0 and a standard deviation of 1 before analysis. These PGSs were chosen because of the substantial genetic overlaps among BD, SCZ, and MDD (Bigdeli et al., 2022; Cross-Disorder Group of the Psychiatric Genomics et al., 2013; Lichtenstein et al., 2009). Moreover, SGAs are used for treatment and prevention of psychosis in both BD and SCZ; hence, the expectation of potential correlations between SGA treatment response in BD participants and genetic variants associated with BD and SCZ. Conversely, we did not expect a significant genetic correlation between SGA treatment response in BD and MDD PGS.

## RESULTS

The cohort included 2,159 adults with BD: 1416 (66%) with BD-I, 691 (32%) with BD-II, and 51 (2%) with schizoaffective BD. The mean age of the cohort was 41.8 years, with 62% female, 84% white, and 14% Hispanic participants (Table 1). SGAs and FGAs were prescribed to 48% and 0.7% of patients (currently), respectively. Tardive dyskinesia (TD) was reported by 11% (66/620) of the cohort, where the TD data was available.

### Comparison between patients currently on SGAs (*n* = 1045) and those not on antipsychotics (*n* = 1114)

There were no significant differences in the rates of BD-I (67% vs. 64%) and BD-II (31% vs. 33%), sex (female: 63% vs. 60%, *p* = 0.07), or race (white: 84% vs. 84%) between patients currently on SGA and currently those not on antipsychotics (Table 1). However, patients on SGA were younger (41.2 ± 14.7 vs. 42.5 ± 15.3 years, *p* = 0.04), more likely to be Hispanic (14% vs. 11%, *p* = 0.047), had a higher body mass index (BMI; 30.4 ± 7.6 vs. 29.5 ± 7.1, *p* = 0.005), and were less likely to be married (42% vs. 49%, *p* < 0.001) and full-time employed (23% vs. 29%, *p* = 0.003), while education levels were similar.

Compared to those not on antipsychotics, current SGA users were more likely to have concomitant MSAC prescriptions (57% vs. 51%, *p* = 0.003), but did not meet the threshold of p<0.001. They also had higher rates of lifetime AN (7% vs. 4%, *p* = 0.003), BN (7% vs. 4%, *p* = 0.003), BED (14% vs. 11%, *p* = 0.03), and a higher EDDS score (−0.03 ± 0.54 vs. −0.12 ± 0.52, *p* < 0.001). History of manic psychosis was more frequent in the current SGA group (29% vs. 17%, *p* < 0.001), as were opioid (13% vs. 9%, *p* = 0.002) and benzodiazepine (11% vs. 6%, *p* = 0.002) use disorders. Medical comorbidities did not differ between groups.

### Comparison between participants currently on one SGA (*n*= 967) and those on more than one SGA (*n* = 78)

Among the 1,045 patients currently on SGA, 967 (92.5%) were on one SGA and 78 (7.5%) were on more than one (**Supplementary Table 1**). No significant differences were found in demographic characteristics (all *p* > 0.08) or prescription rates for lithium (29% vs. 37%, *p* = 0.12), MSACs (57% vs. 50%, *p* = 0.20), and antidepressants (45% vs. 49%, *p* = 0.48) between patients on one SGA compared to those on multiple SGAs. Patients on multiple SGA had higher rates of lifetime BN (13% vs. 7%, *p* = 0.04) and prior suicide attempts (50% vs. 37%, *p* = 0.02). Those on more than one SGA also had a higher likelihood of being diagnosed with hypertension (40% vs. 26%, *p* = 0.046) and upper GI comorbidities (38% vs. 25%, *p* = 0.048), although overall MCIRS scores were similar (6.74 ± 6.56 vs 6.22 ± 6.51, *p* = 0.495). However, none of these findings met the conservative threshold of *p* < 0.001. The prevalence of TD was higher in the multiple SGA group compared to the single SGA group (18% vs. 11%), though this difference was not statistically significant (*p* = 0.21), possibly due to the small sample size.

### Comparison between patients currently on SGA (*n* = 1035) and those on FGA (*n* = 16)

Less than 1% of the overall cohort were on FGA. Patients on FGA were more likely to be female (88% vs. 63%, *p* = 0.046) and had a higher rate of prior suicide attempts (62% vs. 38%, *p* = 0.04). (**Supplementary Table 2**). Although the rate of TD was higher in the FGA group (29% vs. 11%, *p* = 0.16), this difference was not statistically significant.

### Comparison between SGA responders (*n* = 174) and non-responders (*n* = 356)

SGA response data was available for 51% of patients (*n* = 530), with 33% (*n* = 174) classified as responders Table 2. Responders were less likely to be White (60% vs. 76%, *p* < 0.001) and more likely to be Hispanic (40% vs. 19%, *p* < 0.001), with lower education levels (beyond high school: 77% vs. 85%, *p* = 0.02). SGA responders had higher Alda A scores for SGA (8.0 ± 1.0 vs. 3.6 ± 2.0, *p* < 0.001). Responders were less likely to be on antidepressants (36% vs. 48%, *p* = 0.006) and had a lower prevalence of relatives with BD (38% vs. 54%, *p* = 0.002), while rates of current lithium (24% vs. 22%, *p* = 0.668) and MSAC use (55% vs. 55%, *p* = 0.980) were similar between groups. In addition, responders had lower rates of rapid cycling, adult ADHD (13% vs. 24%, *p* = 0.005), childhood ADHD (13% vs. 22%, *p* = 0.017), PTSD (21% vs. 32%, *p* = 0.006), GAD (43% vs. 55%, *p* = 0.015), panic disorder (32% vs. 42%, *p* = 0.024), and social anxiety disorder (15% vs. 25%, *p* = 0.010). They also experienced fewer medical comorbidities, with a lower mean MCIRS score (5.3 ± 6.4 vs. 7.4 ± 7.6, *p* = 0.001).

### GWAS of SGA treatment response in BD patients

**Figure 1:**
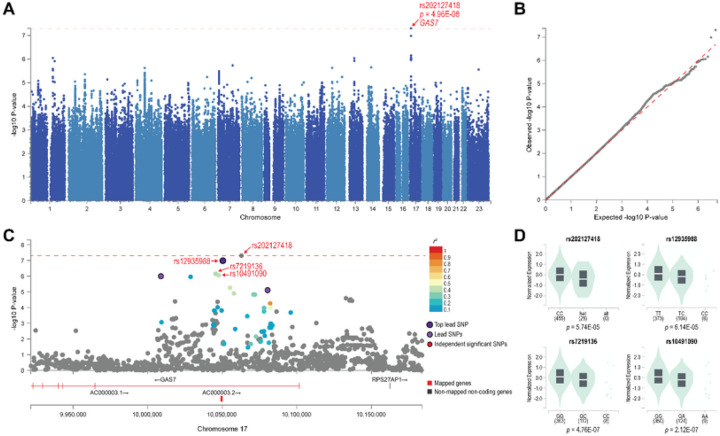
(**A**) Manhattan plot and (**B**) Q-Q plot of second-generation antipsychotics (SGA) treatment response GWAS. One SNP, rs2020127418, attains genome-wide significant association. (**C**) Locus zoom plot of chromosome region around the top SNP. (**D**) The top four SNPs are expression quantitative loci (eQTL) of *GAS7* gene expression in cultured fibroblasts (retrieved from GTEx Portal v.8 on 9/16/2024). Red dotted horizontal lines mark the genome-wide statistical significance threshold *p* = 5E-08.

We performed a GWAS of SGA response among the 517 participants with an Alda score for SGAs while adjusting for sex, genotyping batch, and the first three genomic PCs. We detected a genome-wide significant association between a SNP located in 17p13.1 and SGA Alda A score (rs202127418; *β* = −2.998; *p* = 4.96E-08; minor allele frequency = 0.023; Fig. 1A). Multiple SNPs in this genetic locus with high linkage disequilibrium with the top SNP also showed associations at *p* < 5E-06 and are located in *GAS7* introns (Fig. 1C). The top 4 SNPs are expression quantitative trait loci (eQLT) of *GAS7* gene expression in cultured fibroblasts according to GTEx Portal v.8 (https://gtexportal.org/; Fig. 1D). Top GWAS results are shown in **Supplementary Table 3**.

### Associations of psychiatric disorder PGSs and SGA treatment response for BD, MDD, and SCZ

We did not find significant associations between SGA Alda A score and the PGSs of BD, MDD, or SCZ (FDR > 0.05; **Supplementary Table 4**).

## DISCUSSION

SGA are among the most prescribed medications for BD (Vieta et al., 2018). This study is the first to investigate clinical and biological (genomic) predictors of SGA prescriptions and response in patients with BD. Nearly 50% of individuals with BD were prescribed SGA and less than 1% FGA, aligning with data from the GBC survey and North American cohorts, as well as evidence-based guidelines (Keramatian et al., 2023; Singh et al., 2024). About 8% of patients were on multiple SGA, likely reflecting higher severity burden although the group was too small to formally test this. TD rates were higher in patients prescribed FGAs (29%) compared to those on SGAs (11%), though the difference was not statistically significant, perhaps due to the small sample size. The higher TD rate with FGA is consistent with the literature (Correll and Schenk, 2008), attributed to FGAs’ potent D2 receptor blocking effects.

Several clinical predictors were associated with SGA use, including a history of manic psychosis, higher EDDS scores, and prior suicide attempts. Previous studies have shown that SGA not only increase BMI but may also contribute to disordered eating behaviors (de Beaurepaire, 2021). In our sample, higher EDDS scores among patients receiving SGA suggest a potential association between SGA use and eating disorder symptoms. This could likely be due to SGA, however, given the cross-sectional nature of our data, this finding should be interpreted with caution. Patients on SGA were more likely to use concomitant mood stabilizers (trend level, p = 0.003) but showed no differences in medical comorbidities compared to those not on antipsychotics. SGA responders had lower levels of medical comorbidities and psychiatric comorbidities—such as rapid cycling, ADHD, and PTSD—which have been associated with increased treatment refractoriness in prior studies (Chopra et al., 2024; Roosen and Sienaert, 2022; Salvi et al., 2021).

BD pharmacogenomic studies continued to face challenges due to small sample sizes, in part because of lack of standardized measures for assessing medication treatment response, which hinders data harmonization across cohorts. The Alda Scale, developed and validated for evaluating lithium treatment response (Manchia et al., 2013), demonstrated the value of using a unified instrument across international sites. It also exhibits promise to be applied for other BD medications, such as MSAC (Ho et al., 2025; Ho et al., 2020), enabling within-participant comparison of medication treatment responses. Therefore, the development of a standardized treatment response research instrument would provide a foundation for expanding sample sizes for BD pharmacogenomic research.

In this first GWAS of SGA response in BD, we found one locus in *GAS7* associated with SGA treatment response. *GAS7* encodes growth arrest-specific 7, a member of the growth arrest-specific genes expressed in terminally differentiated cells (Brenner et al., 1989; Schneider et al., 1988). Its most well-known function is neurogenesis via promoting actin filament formation (Gotoh et al., 2013; Ju et al., 1998; Khanal et al., 2023; She et al., 2002; You and Lin-Chao, 2010; Zhang et al., 2016). Reduced *Gas7* expression inhibits neurite formation (Ju et al., 1998; You and Lin-Chao, 2010), while overexpressing *Gas7* increases dendritic spine density (Khanal et al., 2023), suggesting that GAS7 plays an important role in neurodevelopment and synaptic plasticity. *GAS7* has also been implicated in SCZ (Zhang et al., 2016) and Alzheimer’s disease (AD) (Akiyama et al., 2009; Hidaka et al., 2012). In a brain imaging GWAS, *GAS7* was identified among the genes associated with the volume of temporal lobe, a brain region implicated in both SCZ and AD (Kohannim et al., 2012). Interestingly, *GAS7* is among the genes with altered DNA methylation patterns in blood cells of clozapine-treated patients with psychosis compared to psychopharmacotherapeutic-naïve patients with psychosis (Pérez-Aldana et al., 2022). While *GAS7* has not been previously associated with BD, a recent transcriptomic study revealed that it is one of the top upregulated genes in the peripheral blood of patients with BD compared to controls (Torsvik et al., 2023). However, that study had not adjusted for the use of antipsychotics and other medications (Torsvik et al., 2023). Considering this alongside our GWAS findings that the top four SNPs associated with SGA treatment response are eQTLs of *GAS7* expression in cultured fibroblasts, *GAS7* gene expression may potentially be involved in SGA treatment response in BD patients, warranting futher investigation.

Our PGS analysis did not reveal any significant associations between SGA treatment response among participants with BD and the PGSs of BD and SCZ. Previous GWAS studies in SCZ have reported inconsistent findings, with high PGS associated with a range of outcomes—from lower likelihood of improvement with antipsychotics (Zhang et al., 2019) to higher odds of treatment response (De Pieri et al., 2024), as well as increased treatment resistance (Werner et al., 2020). Our null findings suggest that SGA may operate differently in patients with BD and SCZ with respect to psychosis treatment and prevention and treatment of mania/hypomania in BD. They also imply that SGA actions may not directly target the molecular bases of mania/hypomania and psychosis in BD, thus warranting further investigation focused on a better understanding of the biological underpinnings of BD etiology and the development of novel pharmacotherapeutics. Evaluating PGS in relation to specific medications and distinct symptom domains — such as psychosis, activation, and sleep — could represent a promising direction for future research.

### Strength and limitations

Our study has several strengths, along with certain limitations. As one of the largest studies examining clinical predictors of SGA response among patients with BD, it offers valuable insights. A particular strength of the MCBDB is its comprehensive clinical phenotyping.

A few limitations warrant attention. First, there is the potential for recall bias when assessing past medication use and psychiatric diagnoses. The cohort’s majority White demographic also limits the generalizability of findings to other populations. In our exploratory GWAS analysis, the limited sample size precluded conducting sex-specific or ancestry-specific analyses; future studies should include these analyses as sample size permits. In addition, many patients on SGA were concurrently prescribed other medications, which may have impacted the results. Lastly, as this is an observational study, the potential for confounding factors cannot be entirely ruled out.

## Conclusions

In conclusion, approximately 50% of patients with BD are prescribed SGAs, with several clinical predictors identified, including higher BMI, a history of eating disorders, manic psychosis, and opioid/benzodiazepine use disorders. These factors suggest a cohort with greater severity and potential co-prescription of MSACs. Our study also found significant associations between SNPs in *GAS7A* and SGA treatment response in BD, but these findings need to be validated in larger and more ancestry-diverse samples. While PGS analysis did not indicate significant genetic correlations with BD, MDD, and SCZ, *GAS7A*’s involvement in SCZ and Alzheimer’s disease highlights a promising novel candidate for further research.

## Supplementary Files

This is a list of supplementary files associated with this preprint. Click to download.

- SinghSGABipolarDisorderIntJBDSupplTablesAH250714.xlsx

## Figures and Tables

**Table 1.** Comparison of demographic and clinical variables between BD patients currently on SGA versus currently not on any antipsychotics.

	Total (*N*=2159)	No Current Antipsychotics (*N*=1114)	Current SGA (*N*=1045)	*p*-value
**BD type, *n***	2158	1114	1044	
Bipolar I, *n* (%)	1416 (66%)	718 (64%)	698 (67%)	0.324
Bipolar II, *n* (%)	691 (32%)	372 (33%)	319 (31%)	
Schizoaffective, *n* (%)	51 (2%)	24 (2%)	27 (3%)	
**Rapid cycling, *n***	2024	1052	972	
Yes, *n* (%)	1142 (56%)	600 (57%)	542 (56%)	0.564
**Age of BD diagnosis, *n***	2025	1044	981	
≤ 19 years old, *n* (%)	764 (38%)	407 (39%)	357 (36%)	0.229
**Mean age at enrollment** (SD)	41.8 (15.0)	42.5 (15.3)	41.2 (14.7)	0.040
**Female, *n*** (%)	1328 (62%)	665 (60%)	663 (63%)	0.074
**Race, *n***	2146	1106	1040	
White, *n* (%)	1800 (84%)	927 (84%)	873 (84%)	0.351
Black, *n* (%)	56 (3%)	25 (2%)	31 (3%)	
Asian, *n* (%)	24 (1%)	16 (1%)	8 (1%)	
Other, *n* (%)	266 (12%)	138 (12%)	128 (12%)	
**Hispanic ethnicity, *n***	2086	1071	1015	
Yes, *n* (%)	259 (12%)	118 (11%)	141 (14%)	0.047
**Body mass index, *n***	2045	1057	988	
Mean (SD)	29.9 (7.3)	29.5 (7.1)	30.4 (7.6)	0.005
**Currently married, *n***	2064	1056	1008	
Yes, *n* (%)	944 (46%)	522 (49%)	422 (42%)	**<0.001**
**Current full-time employment, *n***	2028	1039	989	
Yes, *n* (%)	522 (26%)	297 (29%)	225 (23%)	0.003
**Highest education level, *n***	2030	1047	983	
High school or less, *n* (%)	58 (3%)	25 (2%)	33 (3%)	0.181
High school graduated, *n* (%)	278 (14%)	134 (13%)	144 (15%)	
Beyond high school graduation, *n* (%)	1694 (83%)	888 (85%)	806 (82%)	
**Current Use of Other BD Medications**				
**Current lithium, *n*** (%)	611 (28%)	303 (27%)	308 (29%)	0.241
**Current MSACs, *n*** (%)	1158 (54%)	563 (51%)	595 (57%)	0.003
**Current antidepressants, *n*** (%)	967 (45%)	498 (45%)	469 (45%)	0.934
**Family History (First-degree Relative)**				
**BD, *n***	1588	808	780	
Yes, *n* (%)	767 (48%)	396 (49%)	371 (48%)	0.564
**Schizophrenia, *n***	1675	862	813	
Yes, *n* (%)	159 (9%)	79 (9%)	80 (10%)	0.637
**Lifetime Psychiatric Illness History**				
**Adult ADHD, *n***	2097	1087	1010	
Yes, *n* (%)	429 (20%)	224 (21%)	205 (20%)	0.860
**Child ADHD, *n***	2084	1078	1006	
Yes, *n* (%)	332 (16%)	168 (16%)	164 (16%)	0.655
**Post-traumatic stress disorder, *n***	2101	1089	1012	
Yes, *n* (%)	548 (26%)	268 (25%)	280 (28%)	0.111
**General anxiety disorder, *n***	2101	1084	1017	
Yes, *n* (%)	1051 (50%)	539 (50%)	512 (50%)	0.776
**Social anxiety disorder, *n***	2092	1082	1010	
Yes, *n* (%)	457 (22%)	222 (21%)	235 (23%)	0.128
**Obsessive compulsive disorder, *n***	2104	1087	1017	
Yes, *n* (%)	274 (13%)	136 (13%)	138 (14%)	0.471
**Phobia, *n***	1402	721	681	
Yes, *n* (%)	137 (10%)	63 (9%)	74 (11%)	0.180
**Panic, *n***	2101	1086	1015	
Yes, *n* (%)	632 (30%)	317 (29%)	315 (31%)	0.357
**Anorexia, *n***	2107	1088	1019	
Yes, *n* (%)	109 (5%)	41 (4%)*	68 (7%)	0.003
**Bulimia, *n***	2102	1084	1018	
Yes, *n* (%)	122 (6%)	47 (4%)*	75 (7%)	0.003
**Binge eating, *n***	2102	1083	1019	
Yes, *n* (%)	256 (12%)	116 (11%)	140 (14%)	0.034
**Manic psychosis, *n***	2131	1096	1035	
Yes, *n* (%)	488 (23%)	183 (17%)	305 (29%)	**<0.001**
**Suicide Attempt History**				
**Suicide attempt (ever), *n***	2138	1099	1039	
Yes, *n* (%)	724 (34%)	333 (30%)	391 (38%)	**<0.001**
**Lifetime Substance Use Disorder History**				
**Nicotine use disorder, *n***	2104	1085	1019	
Yes, *n* (%)	854 (41%)	419 (39%)	435 (43%)	0.057
**Alcohol use disorder, *n***	2115	1093	1022	
Yes, *n* (%)	848 (40%)	430 (39%)	418 (41%)	0.465
**Cocaine use disorder, *n***	2094	1082	1012	
Yes, *n* (%)	305 (15%)	147 (14%)	158 (16%)	0.189
**Marijuana use disorder, *n***	2108	1090	1018	
Yes, *n* (%)	638 (30%)	313 (29%)	325 (32%)	0.109
**Methamphetamine use disorder, *n***	2101	1085	1016	
Yes, *n* (%)	199 (9%)	94 (9%)	105 (10%)	0.191
**Opioid use disorder, *n***	2091	1081	1010	
Yes, *n* (%)	223 (11%)	93 (9%)	130 (13%)	0.002
**Benzodiazepine use disorder, *n***	1380	711	669	
Yes, *n* (%)	118 (9%)	45 (6%)	73 (11%)	0.002
**Medical Comorbidity**				
**Modified CIRS, *n***	1455	749	706	
Medical comorbidity severity^†^, mean (SD)	6.2 (6.5)	6.2 (6.6)	6.3 (6.5)	0.951
Cardiac, n (%)	202 (14%)	109 (15%)	93 (13%)	
Hypertension, n (%)	387 (27%)	195 (26%)	192 (27%)	
Vascular, n (%)	148 (10%)	70 (9%)	78 (11%)	
Respiratory, n (%)	357 (25%)	180 (24%)	177 (25%)	
Eyes, ears, nose, throat, larynx, n (%)	403 (28%)	212 (28%)	191 (27%)	
Upper gastrointestinal, n (%)	369 (25%)	189 (25%)	180 (25%)	
Lower gastrointestinal, n (%)	313 (22%)	169 (23%)	144 (20%)	
Hepatic, n (%)	77 (5%)	40 (5%)	37 (5%)	
Renal, n (%)	134 (9%)	68 (9%)	66 (9%)	
Other genitourinary, n (%)	297 (20%)	157 (21%)	140 (20%)	
Musculoskeletal, integumentary, n (%)	536 (37%)	285 (38%)	251 (36%)	
Neurological, n (%)	548 (38%)	277 (37%)	271 (38%)	
Endocrine-metabolic, n (%)	439 (30%)	214 (29%)	225 (32%)	
**EDDS score**	1807	927	880	**< 0.001**
Mean (SD)	−0.07 (0.53)	−0.12 (0.52)	−0.03 (0.54)	
**Tardive dyskinesia, *n***	620	256	364	
Yes, *n* (%)	66 (11%)	24 (9%)	42 (12%)	0.390

† The mean of the scores of the first 13 categories (excluding psychiatric) in the Modified Cumulative Illness Ration Scale (CIRS).

ADHD: attention-deficit/hyperactivity disorder; BD: bipolar disorder; CIRS: Cumulative Illness Ration Scale; FGA: first-generation antipsychotics; MSAC: Mood-stabilizing anticonvulsant; SGA: second-generation antipsychotics

**Bold *p*-value:** < 0.001;

* Frequency < 5%

**Table 2.** Comparison of demographic and clinical variables between SGA responders (Alda A score ≥ 7) and SGA non-responders (Alda A score < 7).

	Total (*N*=530)	SGA non-responder (*N*=356)	SGA responder (*N*=174)	*p*-value
**BD type, *n***	530	356	174	
Bipolar I, *n* (%)	361 (68%)	248 (70%)	113 (65%)	0.143
Bipolar II, *n* (%)	160 (30%)	100 (28%)	60 (34%)	
Schizoaffective, *n* (%)	9 (2%)	8 (2%)	1 (1%)	
**Rapid cycling, *n***	512	343	169	0.007
Yes, *n* (%)	315 (62%)	225 (66%)	90 (53%)	
**Age of BD diagnosis, *n***	518	346	172	
≤ 19 years old, *n* (%)	229 (44%)	151 (44%)	78 (45%)	0.713
**Age at enrollment, *n***	530	356	174	
Mean (SD)	39.8 (14.6)	40.1 (14.5)	39.2 (14.7)	0.525
**Sex, *n***	530	356	174	
Male, *n* (%)	173 (33%)	119 (33%)	54 (31%)	0.581
Female, *n* (%)	357 (67%)	237 (67%)	120 (69%)	
**Race, *n***	527	354	173	
White, *n* (%)	372 (71%)	269 (76%)	103 (60%)	**<0.001**
Black, *n* (%)	26 (5%)	21 (6%)	5 (3%)	
Asian, *n* (%)	6 (1%)	4 (1%)	2 (1%)	
Other, *n* (%)	123 (23%)	60 (17%)	63 (36%)	
**Hispanic, *n***	507	339	168	
Yes, *n* (%)	130 (26%)	63 (19%)	67 (40%)	**<0.001**
**Body mass index, *n***	504	343	161	
Mean (SD)	30.6 (7.7)	30.9 (7.7)	30.1 (7.8)	0.240
**Currently married, *n***	505	339	166	
Yes, *n* (%)	198 (39%)	138 (41%)	60 (36%)	0.324
**Current full-time employment, *n***	500	335	165	
Yes, *n* (%)	107 (21%)	66 (20%)	41 (25%)	0.187
**Highest education level, *n***	497	334	163	
High school or less, *n* (%)	17 (3%)	7 (2%)	10 (6%)	0.020
High school graduated, *n* (%)	70 (14%)	42 (13%)	28 (17%)	
Beyond high school graduation, *n* (%)	410 (82%)	285 (85%)	125 (77%)	
**BD Medication Response**				
**FGA: Alda A score, *n***	42	28	14	
Mean (SD)	2.7 (2.6)	2.1 (2.3)	3.9 (3.0)	0.044
**SGA: Alda A score, *n***	530	356	174	
Mean (SD)	5.0 (2.7)	3.6 (2.0)	8.0 (1.0)	**<0.001**
**Current Use of Other BD Medications**				
**Current lithium, *n***	530	356	174	
Yes, *n* (%)	119 (22%)	78 (22%)	41 (24%)	0.668
**Current MSACs, *n***	530	356	174	
Yes, *n* (%)	292 (55%)	196 (55%)	96 (55%)	0.980
**Current antidepressants, *n***	530	356	174	
Yes, *n* (%)	234 (44%)	172 (48%)	62 (36%)	0.006
**Family History (First-degree Relative)**				
**BD, *n***	408	267	141	
Yes, *n* (%)	197 (48%)	144 (54%)	53 (38%)	0.002
**Schizophrenia, *n***	426	278	148	
Yes, *n* (%)	37 (9%)	29 (10%)	8 (5%)	0.079
**Lifetime Psychiatric Illness History**				
**Adult ADHD, *n***	520	349	171	
Yes, *n* (%)	107 (21%)	84 (24%)	23 (13%)	0.005
**Child ADHD, *n***	518	349	169	
Yes, *n* (%)	98 (19%)	76 (22%)	22 (13%)	0.017
**Post-traumatic stress disorder, *n***	515	345	170	
Yes, *n* (%)	146 (28%)	111 (32%)	35 (21%)	0.006
**General anxiety disorder, *n***	519	348	171	
Yes, *n* (%)	264 (51%)	190 (55%)	74 (43%)	0.015
**Social anxiety disorder, *n***	518	348	170	
Yes, *n* (%)	114 (22%)	88 (25%)	26 (15%)	0.010
**Obsessive compulsive disorder, *n***	519	348	171	
Yes, *n* (%)	68 (13%)	52 (15%)	16 (9%)	0.076
**Phobia, *n***	295	211	84	
Yes, *n* (%)	32 (11%)	26 (12%)	6 (7%)	0.197
**Panic, *n***	520	349	171	
Yes, *n* (%)	203 (39%)	148 (42%)	55 (32%)	0.024
**Anorexia, *n***	520	350	170	
Yes, *n* (%)	24 (5%)*	19 (5%)	5 (3%)*	0.205
**Bulimia, *n***	520	350	170	
Yes, *n* (%)	30 (6%)	21 (6%)	9 (5%)	0.746
**Binge eating, *n***	519	349	170	
Yes, *n* (%)	105 (20%)	67 (19%)	38 (22%)	0.401
**Psychosis history, *n***	522	349	173	
Yes, *n* (%)	248 (48%)	157 (45%)	91 (53%)	0.101
**Manic psychosis, *n***	522	349	173	
Yes, *n* (%)	143 (27%)	88 (25%)	55 (32%)	0.113
**Suicide Attempt History**				
**Suicide attempt (ever), *n***	524	352	172	
Yes, *n* (%)	214 (41%)	148 (42%)	66 (38%)	0.422
**Lifetime Substance Use Disorder History**				
Nicotine use disorder, *n*	517	348	169	
Yes, *n* (%)	181 (35%)	129 (37%)	52 (31%)	0.159
**Alcohol use disorder, *n***	519	348	171	
Yes, *n* (%)	186 (36%)	117 (34%)	69 (40%)	0.133
**Cocaine use disorder, *n***	518	347	171	
Yes, *n* (%)	60 (12%)	38 (11%)	22 (13%)	0.522
**Marijuana use disorder, *n***	521	350	171	
Yes, *n* (%)	127 (24%)	84 (24%)	43 (25%)	0.775
**Methamphetamine use disorder, *n***	521	349	172	
Yes, *n* (%)	31 (6%)	18 (5%)	13 (8%)	0.276
**Opioid use disorder, *n***	517	346	171	
Yes, *n* (%)	45 (9%)	36 (10%)	9 (5%)	0.051
**Benzodiazepine use disorder, *n***	363	237	126	
Yes, *n* (%)	33 (9%)	23 (10%)	10 (8%)	0.577
**Medical Comorbidity**				
**Modified CIRS, *n***	301	214	87	
Medical comorbidity severity^†^, mean (SD)	6.7 (7.3)	7.4 (7.6)	5.3 (6.4)	**0.001**
Cardiac, n (%)	50 (17%)	41 (19%)	9 (10%)	
Hypertension, n (%)	98 (33%)	69 (32%)	29 (33%)	
Vascular, n (%)	37 (12%)	28 (13%)	9 (10%)	
Respiratory, n (%)	105 (35%)	77 (36%)	28 (32%)	
Eyes, ears, nose, throat, larynx, n (%)	116 (39%)	77 (36%)	39 (45%)	
Upper gastrointestinal, n (%)	112 (37%)	87 (41%)	25 (29%)	
Lower gastrointestinal, n (%)	77 (26%)	54 (25%)	23 (26%)	
Hepatic, n (%)	14 (5%)	7 (3%)	7 (8%)	
Renal, n (%)	31 (10%)	24 (11%)	7 (8%)	
Other genitourinary, n (%)	77 (26%)	54 (25%)	23 (26%)	
Musculoskeletal, integumentary, n (%)	146 (49%)	112 (52%)	34 (39%)	
Neurological, n (%)	150 (50%)	113 (53%)	37 (43%)	
Endocrine-metabolic, n (%)	119 (40%)	86 (40%)	33 (38%)	
**EDDS score**	442	299	143	0.253
Mean (SD)	−0.02 (0.53)	−0.06 (0.51)	0.00 (0.53)	
**Tardive dyskinesia, *n***	238	149	89	
Yes, *n* (%)	23 (10%)	16 (11%)	7 (8%)	0.468

† The mean of the scores of the first 13 categories (excluding psychiatric) in the Modified Cumulative Illness Ration Scale (CIRS).

ADHD: attention-deficit/hyperactivity disorder; BD: bipolar disorder; CIRS: Cumulative Illness Ration Scale; FGA: first-generation antipsychotics; MSAC: Mood-stabilizing anticonvulsant; SGA: second-generation antipsychotics

**Bold *p*-value:** < 0.001

* Frequency < 5%

## Data Availability

The datasets used and/or analyzed during the current study are available from the corresponding author on reasonable request.
